# A transgenic zebrafish model for the *in vivo* study of the blood and choroid plexus brain barriers using *claudin 5*

**DOI:** 10.1242/bio.030494

**Published:** 2018-02-15

**Authors:** Lisanne Martine van Leeuwen, Robert J. Evans, Kin Ki Jim, Theo Verboom, Xiaoming Fang, Aleksandra Bojarczuk, Jarema Malicki, Simon Andrew Johnston, Astrid Marijke van der Sar

**Affiliations:** 1 Department of Medical Microbiology & Infection control, VU Medical Center, Amsterdam 1081HV, The Netherlands; 2 Department of Pediatric Infectious Diseases & Immunology, VU Medical Center, Amsterdam 1007MB, The Netherlands; 3 Bateson Centre, University of Sheffield, Sheffield, S10 2TN, United Kingdom; 4 Department of Infection, Immunity and Cardiovascular Disease, Medical School, University of Sheffield, Sheffield, S10 2TN, United Kingdom; 5 Department of Biomedical Sciences, University of Sheffield, Sheffield, S10 2TN, United Kingdom

**Keywords:** Claudin 5, Tight junction, Zebrafish, Blood brain barrier, Choroid plexus, Transgene

## Abstract

The central nervous system (CNS) has specific barriers that protect the brain from potential threats and tightly regulate molecular transport. Despite the critical functions of the CNS barriers, the mechanisms underlying their development and function are not well understood, and there are very limited experimental models for their study. Claudin 5 is a tight junction protein required for blood brain barrier (BBB) and, probably, choroid plexus (CP) structure and function in vertebrates. Here, we show that the gene *claudin 5a* is the zebrafish orthologue with high fidelity expression, in the BBB and CP barriers, that demonstrates the conservation of the BBB and CP between humans and zebrafish. Expression of *claudin 5a* correlates with developmental tightening of the BBB and is restricted to a subset of the brain vasculature clearly delineating the BBB. We show that *claudin 5a*-expressing cells of the CP are ciliated ependymal cells that drive fluid flow in the brain ventricles. Finally, we find that CP development precedes BBB development and that *claudin 5a* expression occurs simultaneously with angiogenesis. Thus, our novel transgenic zebrafish represents an ideal model to study CNS barrier development and function, critical in understanding the mechanisms underlying CNS barrier function in health and disease.

## Abstract (precis)

**Summary:** A novel transgenic zebrafish, using *claudin 5a*, represents an ideal model to study blood brain barrier and choroid plexus barrier development and function *in vivo*.

## INTRODUCTION

The central nervous system (CNS) is protected by three specialized barriers that shield the vulnerable brain tissue from potential threats and actively regulate exchange of ions and nutrients. The blood brain barrier (BBB) is formed by endothelial cells between blood and brain interstitial fluid and has extensive control over the immediate microenvironment of the CNS (Abbott et al., 2006, 2010). Less studied are the blood-cerebrospinal fluid (CSF) barrier, formed by the epithelial cell layer of the choroid plexus (CP) between blood and ventricular CSF, and the epithelial cell layer of the meningeal arachnoid between blood and subarachnoid CSF (Abbott et al., 2006, 2010; Obermeier et al., 2013).

The BBB and blood-CSF barrier tissues have tight junctions (TJs), consisting of protein complexes that seal adjacent cells and actively regulate barrier integrity (Greene and Campbell, 2016). TJs are protein complexes containing occludins and claudins that provide a physical barrier to block free paracellular diffusion of solutes and macromolecules (Abbott et al., 2010). More than 20 different claudin isoforms are known, of which at least four, Claudin 1, 3, 5 and 12, are involved in establishing and regulating TJs in mammalian brain endothelial cells (Abdelilah-Seyfried, 2010; Greene and Campbell, 2016). Of these, *claudin 5* is the most strongly expressed in mammalian brain microvessels (Zhang et al., 2012). Although this protein was shown to be important for barrier integrity in mice, the expression of *claudin 5* is not conserved in the murine CP and is not a definitive marker of the BBB (Nitta et al., 2003).

Links with BBB breakdown or dysfunction have been shown for neurodegenerative processes, inflammation and infection. These include, but are not limited to, Alzheimer's disease (Schenk and de Vries, 2016; Zenaro et al., 2016), multiple sclerosis (Macrez et al., 2016; Schenk and de Vries, 2016), amyotrophic lateral sclerosis (Garbuzova-Davis and Sanberg, 2014; Garbuzova-Davis et al., 2012), vascular dementia (Ueno et al., 2016), autoimmune encephalitis (Platt et al., 2017) and infectious meningoencephalitis (Coureuil et al., 2017; Gibson and Johnston, 2015; Swanson and McGavern, 2015). Targeting the breakdown or dysfunction of the BBB in CNS disease has significant potential as treatment target, but is severely hampered by a lack of experimental models and current treatment is limited to broad spectrum immunosuppression/anti-inflammatory treatment with, for example, glucocorticosteroids (Obermeier et al., 2013). Furthermore, to improve drug delivery in CNS disease, specific modulation of therapeutic delivery to the brain with limited neurotoxicity is critical (Greene and Campbell, 2016), but there are very limited experimental models that allow the required noninvasive imaging and mechanistic studies (Greene and Campbell, 2016; O'Keeffe and Campbell, 2016; Zhang et al., 2010).

*In vitro* experimental models exist to study different aspects of the BBB, but none of them can completely mimic the complex interplay between BBB and other cells, such as immune cells or pathogens. In addition, *in vivo* systems are often restricted by the use of a single method, such as single molecular tracer injections or immunohistochemistry. Moreover, real-time *in vivo* imaging of both the BBB and CP is not possible in current models (Blanchette and Daneman, 2015). Therefore, we set out to develop a new model and demonstrate its potential in understanding the mechanistic biology of the BBB and CP.

The zebrafish (*Danio rerio*) model has proven to be highly accessible for real-time imaging especially in combination with fluorescently labelled tissues, and is therefore extensively used to study development and many aspects of human disease (Holtzman et al., 2016). Recently, two transgenic zebrafish lines were developed to visualize CNS angiogenesis and BBB development *in vivo*, demonstrating that these events occur simultaneously (Umans et al., 2017). The zebrafish BBB and CP are similar to higher vertebrates, and Claudin 5a has been suggested to play an essential role in establishment and maintenance of these barriers between systemic circulation and CNS (Henson et al., 2014; Jeong et al., 2008; Xie et al., 2010). Early in development, at 14 h postfertilization (hpf), *claudin 5a* is expressed in the entire developing zebrafish CNS (Zhang et al., 2010). However, soon after, labelling is confined to the CP and brain vasculature (from 20 hpf and 48 hpf onwards, respectively) (Xie et al., 2010; Zhang et al., 2010). Functional studies have shown size-dependent exclusion of fluorescent tracers injected in the circulation from 2 days postfertilization (dpf) onwards, indicative of the functional maturation of the BBB shortly after TJ formation (Fleming et al., 2013; Jeong et al., 2008; van Leeuwen et al., 2014; Xie et al., 2010). Furthermore, Claudin 5a has been suggested to be involved in the establishment of the neuroepithelial ventricular barrier, which is essential for brain ventricle expansion and subsequent brain development (Zhang et al., 2010, 2012). With the use of several enhancer trap lines the presence of a diencephalic and myelencephalic CP (dCP and mCP, respectively) has been suggested to be present in zebrafish larvae early in development (Bill and Korzh, 2014; Bill et al., 2008; García-Lecea et al., 2008; Henson et al., 2014). Therefore, we considered Claudin 5a to be an excellent candidate as the basis for our new *in vivo* model for the BBB and CP, which adds specificity to the currently existing model systems.

In this study, we have identified *claudin 5a* as the zebrafish gene equivalent to human claudin 5. We have generated a *claudin 5a* reporter transgenic with high fidelity expression in both the BBB and CP, making time-lapse imaging of early development of these structures possible. Using our new transgenic we demonstrate that the CP forms prior to expression of *claudin 5a* in brain blood vessels and validate our model by showing that once the BBB is established *claudin 5a* expression coincides with new BBB vessel formation. In addition, *Claudin 5a* expressing cells in the CP are ciliated ependymal cells that drive fluid flow early in development.

## RESULTS

### Zebrafish *claudin 5a* is the human claudin 5 orthologue

**Fig. 1.**
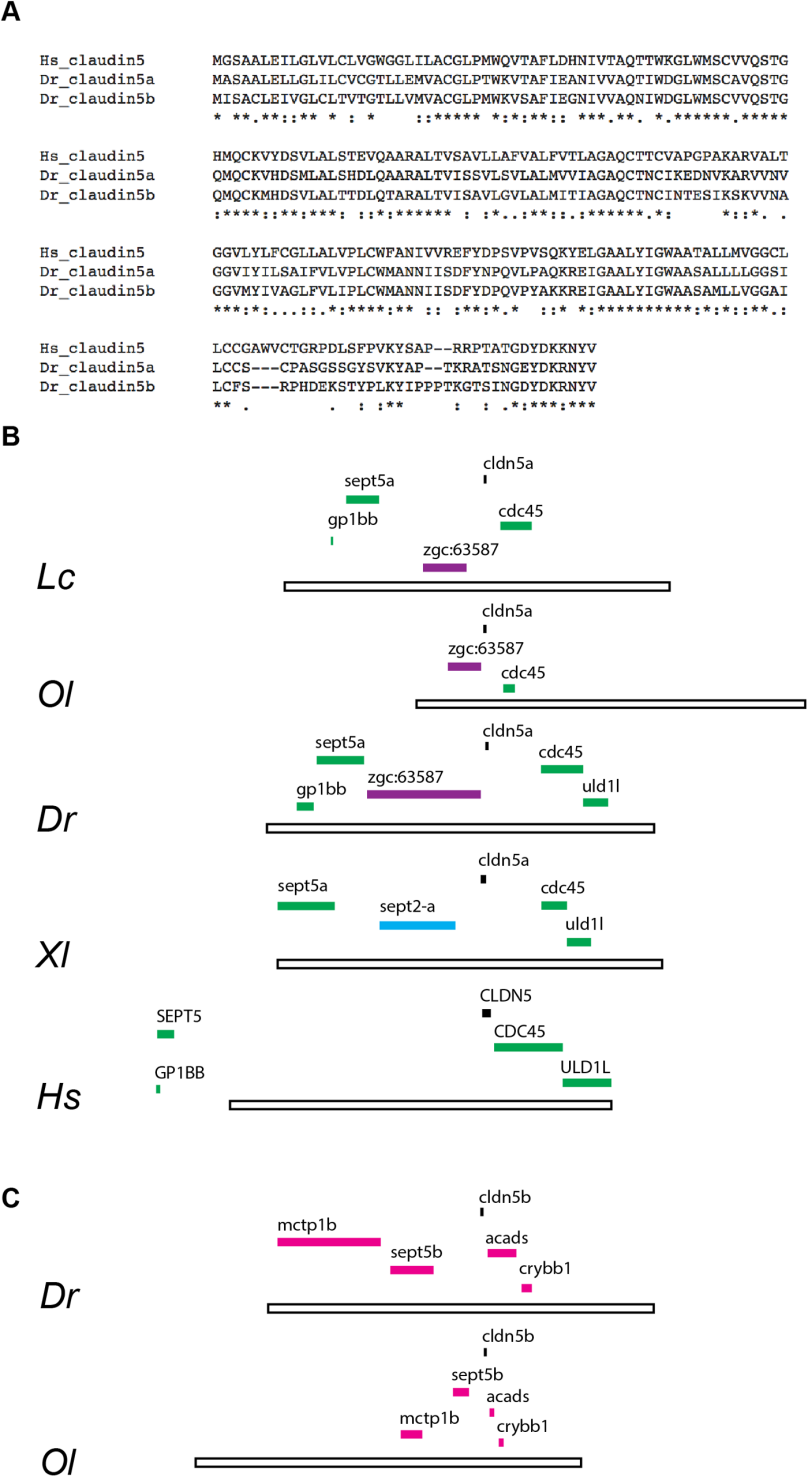
**Zebrafish Claudin 5a is the homologue of human claudin 5.** (A) Protein sequence alignment of human (Hs; *Homo sapiens*) claudin 5, zebrafish (Dr; *Danio rerio*) Claudin 5a and Claudin 5b using Clustal Omega. (B,C) Syntenic analysis of *claudin 5a* (B) and *claudin 5b* (C) genes. (B) Green: Syntenic genes in the *claudin 5a* locus across *Latimeria chalumnae* (Lc), *Oryzias latipes* (Ol), *Danio rerio* (Dr), *Xenopus laevis* (Xl) and *Homo sapiens* (Hs). Purple: Syntenic genes in the *claudin 5a* locus across Osteichthyes (Lc, Ol, Dr). Blue: Unique gene present in the *claudin 5a* locus of *Xenopus laevis*. Open bar, 250 Kb. (C) *Claudin 5b* is only present in Actinopterygii (ray-finned fish), here represented by *Oryzias latipes* (Ol) and *Danio rerio* (Dr), which show conserved synteny. Syntenic genes in the *claudin 5b* locus across Actinopterygii.

In order to identify the zebrafish (Dr) orthologue of human (Hs) protein claudin 5 (*CLDN5* gene) we used a BLASTP search of the zebrafish genome (GRCz10) using the protein sequence of Hs claudin 5. Two zebrafish proteins, Claudin 5a and Claudin 5b, were identified as being most similar by protein sequence (56.9% and 54.8% identical, respectively; Fig. 1A). Alignment of the zebrafish protein sequences to the Hs sequence could not identify which of these two proteins is the orthologue. Examination of the genomic region and closer gene relatives of *claudin 5a* and *5b* clearly showed that *claudin 5a* shared synteny with Hs *CLDN5* and that *claudin 5b* was only present in ray-finned fish (Fig. 1B,C; data not shown). In addition, examination of zebrafish expression patterns (Thisse et al., 2004) showed that *claudin 5a* was expressed in the CNS ventricle region while *claudin 5b* had a cardiovascular patterning. Together, we took this as sufficient evidence that *claudin 5a* was the correct target gene. Using BAC recombineering (Abe et al., 2011) we inserted enhanced green fluorescent protein (EGFP) at the translation start site of the *claudin 5a* gene with ∼200 Kb of flanking sequence to maximise fidelity of EGFP expression to endogenous *claudin 5a* (Fig. S1).

### *Claudin 5a* is expressed in the CP of zebrafish at 1 dpf

**Fig. 2.**
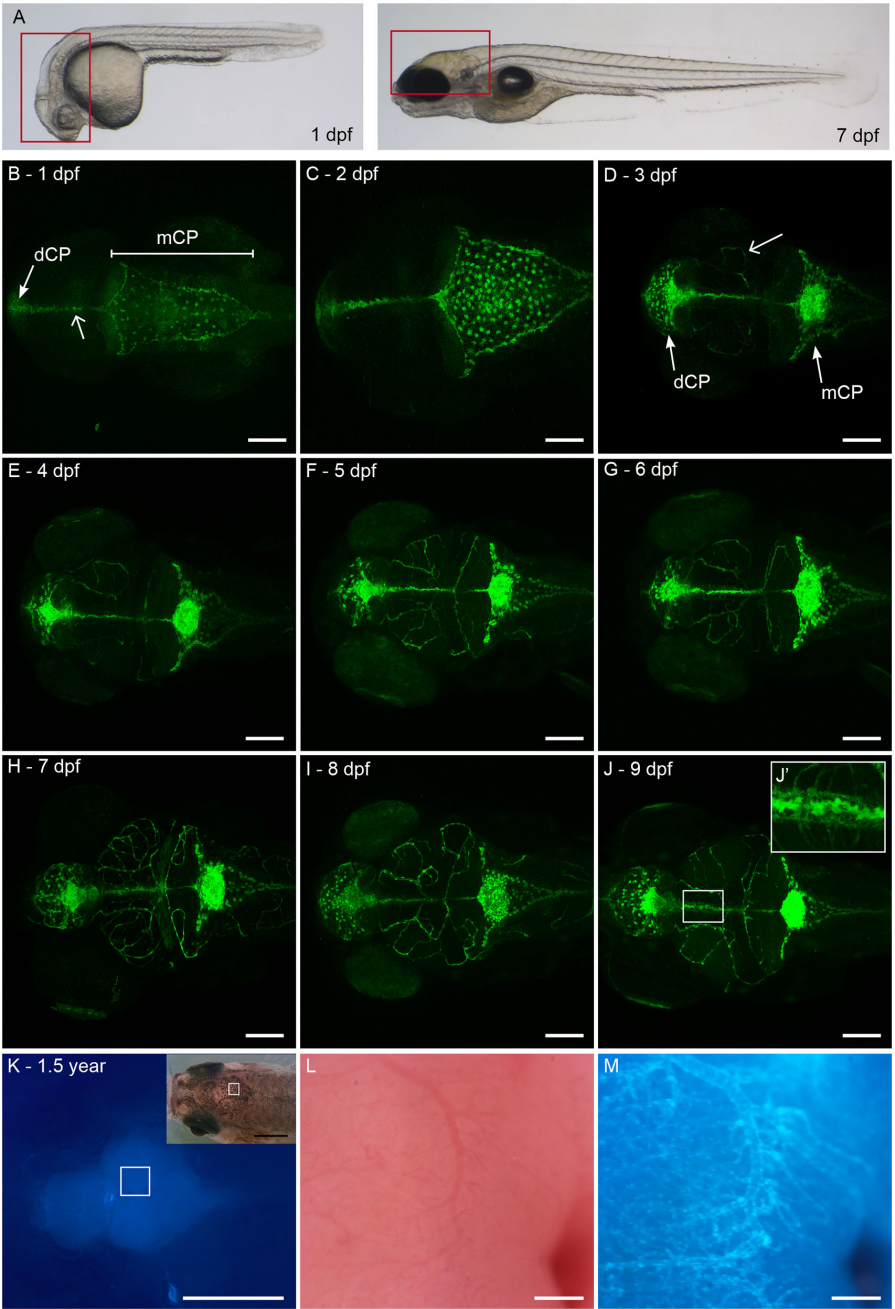
**Developmental expression of *claudin 5a*.** (A) Lateral view of a Casper zebrafish larva at 1 dpf and 7 dpf. Boxed areas represent the brain regions of which confocal images are shown in B-J. (B-J) Z-stacks of dorsal view of larval head to visualize development of GFP expression from 1 to 9 dpf. GFP expression can be found in the dCP and mCP from 1 dpf onwards (B, closed arrow). In addition, labelling is observed in the midline connecting the dCP and mCP (B, open arrow, J’). From 3 dpf onwards, labelling in brain parenchyma is observed (D, open arrow). (K) Diffuse GFP expression in brain region of adult zebrafish, 1.5 years, with corresponding brightfield image. The boxed area in K and its inset is enlarged in M and L, respectively. (L) Brightfield image of blood vessel in adult brain, colocalizing with (M) Claudin 5a:GFP expression. Scale bars: 100 μm in B-J; 1 mm in K; 200 μm in L,M.

To study the developmental expression of *claudin 5a* in *TgBAC(cldn5a:EGFP)^vum1^* larvae and correlate this to previous performed immunohistochemical analysis (Xie et al., 2010; Zhang et al., 2012), we performed noninvasive imaging of the brain region of larvae daily between 1 and 9 dpf and imaging of adult zebrafish at 1.5 years (Fig. 2). As early as 24 hpf, GFP expression was observed in the area of the mCP and dCP (Fig. 2B, arrows). The mCP consisted of a large sheet of cells covering the roof of the hindbrain ventricle early in development (Fig. 2B,C) that developed into a compact cluster located in the midline of the larval head at 3 dpf (Fig. 2D). In addition to expression in both CPs, labelling in brain parenchyma, presumably colocalizing with vasculature, and spinal cord was observed from 3 dpf onwards (Fig. 2D, arrow). Between 3 and 5 dpf, *claudin 5a* expression rapidly expanded in the entire parenchyma (Fig. 2F). Interestingly, strong labelling in the midline of the larval head was observed (Fig. 2B, open arrow). This labelling appeared at the same time in both CPs, connected these structures, and was sustained through development (Fig. 2J,J′). In addition to *Claudin 5a:GFP* expression during development and maturation of the BBB and CP, expression was maintained in the BBB of adult fish at 1.5 years (Fig. 2K-M). Unfortunately, both CPs were difficult to access in whole adult brain, thus CP labelling in adults could not be confirmed. Although Claudin 5a specifically labels CNS barriers in zebrafish, transient expression was observed in the caudal hematopoietic tissue (CHT), the tip of the tail and the heart region (Fig. S2). This expression was only present during early development and disappeared in later larval stages (data not shown). Collectively, our *TgBAC(cldn5a:EGFP)^vum1^* larvae and adult zebrafish showed specific expression in brain vasculature and, in larvae, in both CPs labelling the BBB and blood-CSF barrier, respectively.

### *Claudin 5a* expression in brain vasculature rapidly expands between 3 dpf and 4 dpf

**Fig. 3.**
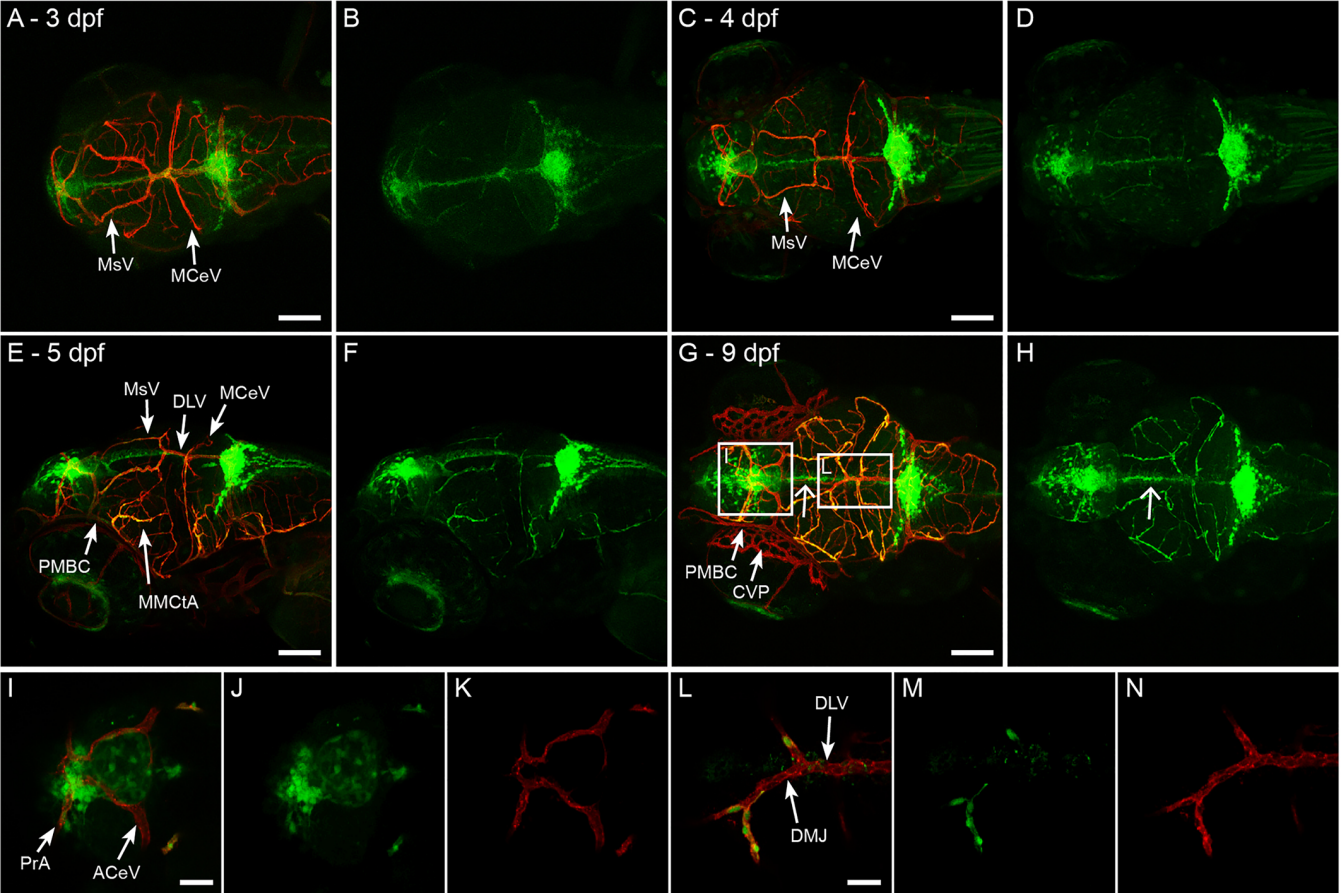
**Development of *Claudin 5a* expression in brain vasculature.** (A,B) Dorsal view of head of *Tg(kdrl:mCherry)^is5^;TgBAC(cldn5a:EGFP)^vum2^* larva at 3 dpf. Merge of both channels is shown in A, single green fluorescent expression is shown in B. *Claudin 5a* is first expressed in the mesencephalic vein (MsV) and middle cerebral vein (MCeV). (C-F) Expansion of *claudin 5a* expression over time, with almost all blood vessels expressing *claudin 5a* at 5 dpf (E-F): dorsal view (C,D), dorsal/lateral view (E,F). (G,H) Dorsal view of head of *Tg(kdrl:mCherry)^is5^;TgBAC(cldn5a:EGFP)^vum2^* larva at 9 dpf. The boxed areas are shown enlarged in I-K and L-N. Open arrow shows the strong expression of claudin 5a in the midline in the absence of a colocalizing blood vessel (H). Specific spots that never express *claudin 5a* are the primordial midbrain channels (PMBC) and choroidal vascular plexus (CVP) (G), anterior cerebral vein (ACeV) (I), and dorsal midline junction (DMJ) and dorsal longitudinal vein (DLV) (L). Scale bars: 100 μm in A,C,E,G; 25 μm in I,L.

To study if Claudin 5a can be found in tight junctions of brain vasculature and therefore represents the BBB, we injected our construct in the vascular specific reporter line *Tg(kdrl:mCherry)^is5^* (Jin et al., 2005) to generate a double transgenic line, *Tg(kdrl:mCherry)^is5^;TgBAC(cldn5a:EGFP)^vum2^*. Using the previously described detailed anatomical description of vasculature development (Isogai et al., 2001), we observed that expression of *claudin 5a* first appeared in the mesencephalic vein (MsV) and middle cerebral vein (MCeV) at 3 dpf (Fig. 3A,B). Subsequent expansion of *claudin 5a* expression between 3 and 4 dpf occurred in large vessels first (Fig. 3C,D). At 5 dpf, nearly all vessels, veins and arteries, show green fluorescence indicating that *claudin 5a* is expressed in virtually all the vessels in the zebrafish brain (Fig. 3E,F). Intriguingly, a certain number of specific areas never showed *claudin 5a* expression (15 of 16 larvae, three biological independent experiments, Fig. 3G): the primordial midbrain channels (PMBC), choroidal vascular plexus (CVP) (Fig. 3G, arrows), anterior cerebral vein frontally located (ACeV) (Fig. 3I-K), and at the location of the midbrain the dorsal midline junction (DMJ) and dorsal longitudinal vein (DLV) (Fig. 3L-N). In addition, the strong labelling in the midline of the larval head did not colocalize with blood vessels (Fig. 3H, open arrow). The Claudin 5a-deficient regions were sustained through development until at least 9 dpf and were present independent of zebrafish background [WT, Casper or *Tg(kdrl:mCherry)*].

### *Claudin 5a* expression occurs prior to new BBB vessel formation

**Fig. 4.**
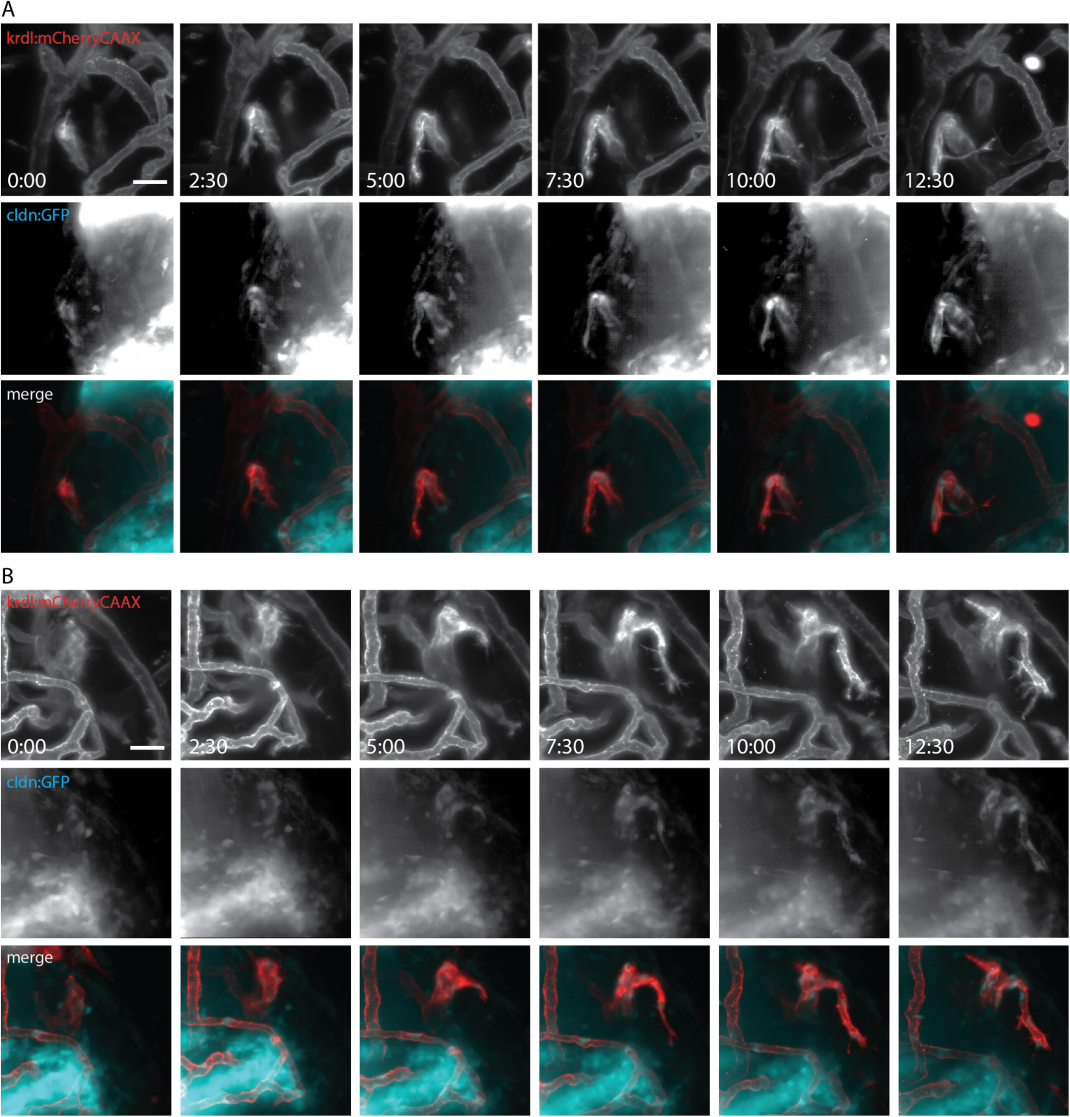
**Initiation of *claudin 5a* expression coincides with blood vessel formation.** (A,B) Still images of two time lapses of blood vessel development in *Tg(kdrl:mCherry)^is5^;TgBAC(cldn5a:EGFP)^vum2^* larva at 96 hpf. Images were taken every 2.5 h for a period of 12.5 h on blood vessels in the optic tectum of the midbrain. Images of single channels show blood vessel development (*kdrl:mcherryCAAX*) on the top row, coinciding with development of claudin 5a in the middle row. Merged images are shown in the bottom row. Scale bars: 20 μm. See corresponding movies of time lapses (Movies 1 and 2).

As stated above, we had identified that Claudin 5a was first present in the larger vessels. Development and expansion of brain vasculature continues after 4 dpf, when *claudin 5a* expression is established. The timing of tight junction protein expression in the BBB has been unstudied for long due to the absence of a suitable *in vivo* model (Haddad-Tóvolli et al., 2017). Recently, two transgenic zebrafish lines were developed that showed that tight junction protein expression occur together with CNS angiogenesis (Umans et al., 2017). Therefore, we aimed to test and validate our model in this respect. Using long time-lapse imaging over 12 h, starting at 96 hpf (3 dpf), in our *Tg(kdrl:mCherry)^is5^;TgBAC(cldn5a:EGFP)^vum2^* double transgenic, we were able to identify sprouting vessels and follow their growth and correlation with *claudin 5a* expression. Careful analysis of sprouting vessels revealed that, in every case, *claudin 5a* expression was observed simultaneously with the initiation of a new vessel (Fig. 4; Movies 1 and 2). This indicates that components of tight junctions were expressed from the initiation of BBB angiogenesis and demonstrates the essential nature of early expression of these junctions in BBB development.

### Zebrafish larvae possess two separate blood-CP barriers that exhibit collective cell migration

**Fig. 5.**
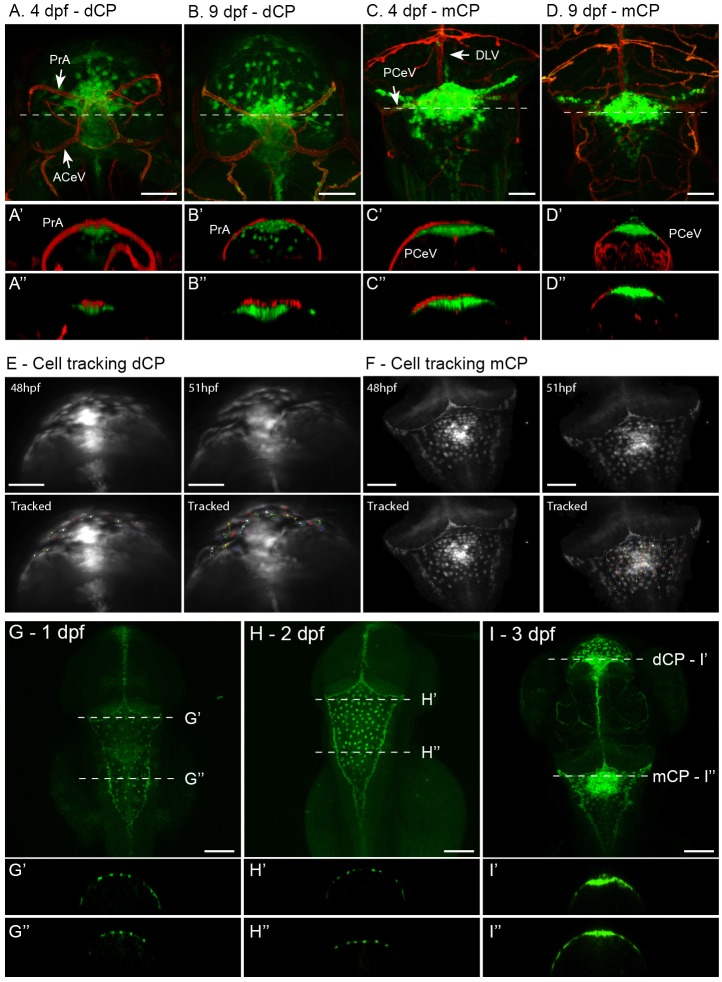
**Blood-CP barrier.** (A) Dorsal view of dCP of *Tg(kdrl:mCherry)^is5^;TgBAC(cldn5a:EGFP)^vum2^* larva at 4 dpf, showing the close correlation between the vasculature (red) and *claudin 5a*-expressing cells (green). The ACeV and PrA form a vascular circuit surrounding the dCP. Transversal view with 3D model (A′) and single Z-slice at the dotted line in A (A″) visualize the close correlation between these structures. (B) Dorsal view of dCP in a larva at 9 dpf, with transversal view in a 3D model (B′) and single Z-slice at dotted line (B″). (C) At 9 dpf, the blood-dCP barrier is formed by the PrA. At the level of the mCP, the PCeV and DLV form a vascular circuit. Transversal view in C′ with a 3D model and C″ with a single Z-slice at the level of the dotted line in C show the close correlation between the mCP and vasculature. (D) Dorsal view of mCP in a larva at 9 dpf, with transversal view in a 3D model (D′) and single Z-slice at dotted line (D″). ACeV, anterior cerebral vein; DLV, dorsal longitudinal vein; PCeV, posterior cerebral vein; PrA, prosencephalic artery. (E) First and last images of time lapse of dCP cell migration at 48-51 dpf. Time lapse is presented in Movie 3. (F) First and last images of time lapse of mCP cell migration at 48-51 dpf. Time lapse is presented in Movie 4. (G-I) Dorsal view of head of *TgBAC(cldn5a:EGFP)^vum2^* larvae between 1 dpf and 3 dpf, the timeframe in which the major morphological transformation is observed. Transversal section is shown for every time point in G′,H′,I′ and G″,H″,I″, corresponding to the dotted lines depicted in G-I. This visualizes the superficial localisation of the GFP-expressing cells of the mCP and dCP. Scale bars: 50 μm in A-F; 100 μm in G-I.

To determine the position of the *claudin 5a*-expressing cells being a major component of the blood-CP barrier, its localisation in respect to the vasculature was analysed in more detail in the double transgenic line *Tg(kdrl:mCherry)^is5^;TgBAC(cldn5a:EGFP)^vum2^*. Three-dimensional confocal analysis revealed that the ACeV and prosencephalic artery (PrA) formed a vascular circuit early in development, which overlapped with *claudin 5a* expression at the location of the dCP (Fig. 5A,B). In the mCP in the roof of the hindbrain ventricle a similar pattern was seen: the DLV and both posterior cerebral veins (PCeV) formed a vascular circuit closely related to the cells expressing *claudin 5a* (Fig. 5C,D). This colocalization did not change during the course of days (Fig. 5A-D, compare 4 dpf with 9 dpf), with the barrier between systemic circulation and CP established early in development. Detailed analysis of the cell dynamics of both CPs identified that the main morphological transformations occur between 1 dpf and 3 dpf (Fig. 5E-I), as proposed by a previous study performed in a CP enhancer trap zebrafish transgenic (Bill et al., 2008; García-Lecea et al., 2008). We performed cell-tracking experiments on both CPs, to demonstrate time-lapse possibilities in this model and reveal mechanisms involved in CP formation. Cell tracking demonstrated that both structures formed via cell migration rather than cell division (Fig. 5E,F; Movies 3 and 4), where *claudin 5a* expressing cells formed a single layer of closely connected cells that were localized in the roof of both ventricles, before and after movement to the midline (Fig. 5G-I).

### *Claudin 5a* expression delineates the structured epithelial sheet of the CP

**Fig. 6.**
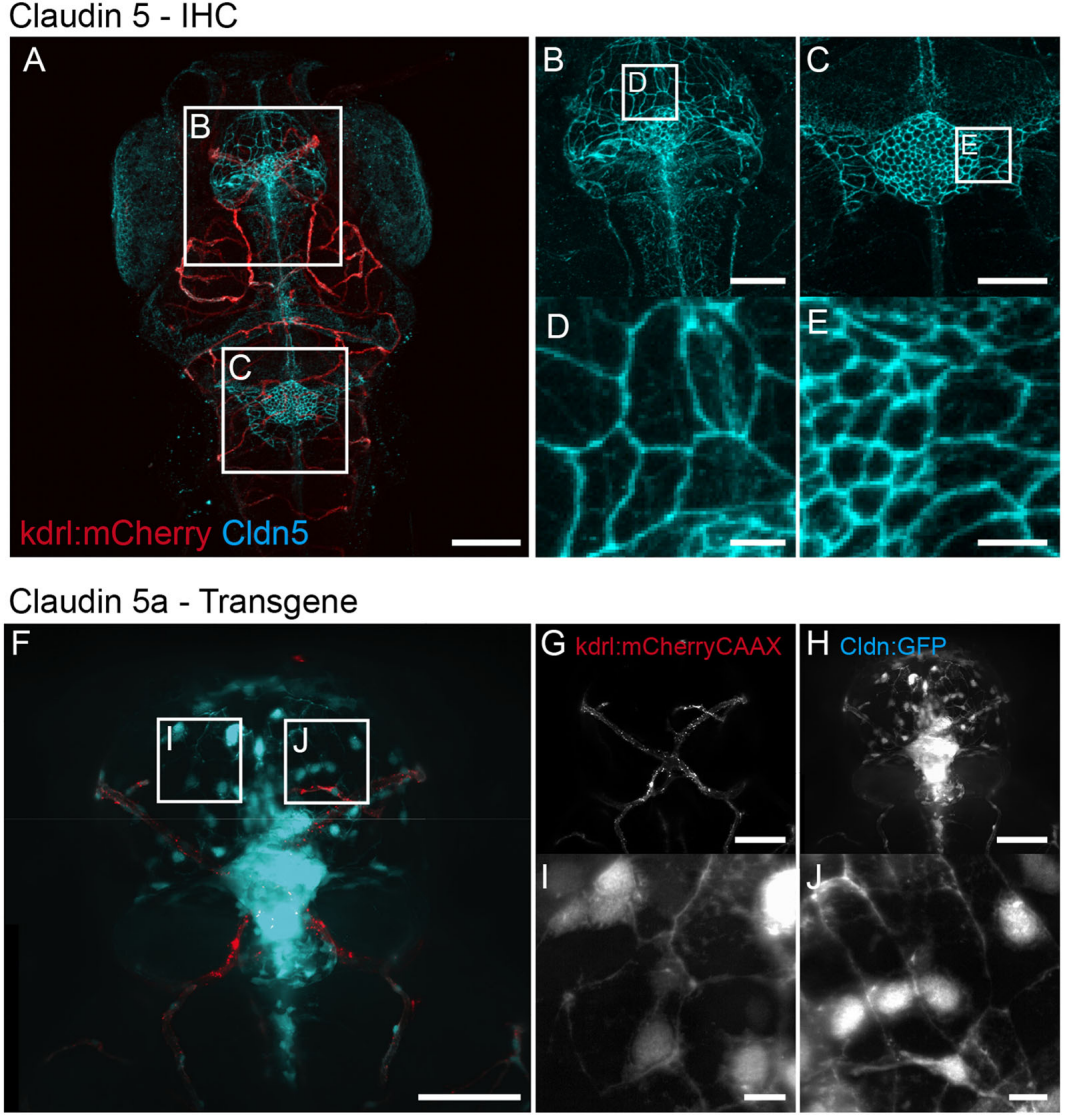
**CP morphology.** (A) Dorsal view of the head of a 7 dpf *Tg(kdrl:mCherry)^is5^* larva showing blood vessels (red) and immunohistochemistry (IHC) labelling of Claudin 5 (cyan). Boxed areas are shown enlarged in B,D and C,E, and show detailed analysis of the collection of cells that forms the dCP (B) and mCP (C) and is closely connected by Claudin 5. The sheet of cells found in the dCP and mCP of a 7 dpf *TgBAC(cldn5a:EGFP)^vum2^* larva is similar to the phenotype in A-E, although subcellular localizations are not the same. (F) Z-stack of mCP of a *Tg(kdrl:mCherry)^is5^;TgBAC(cldn5a:EGFP)^vum2^* larva with red fluorescent blood vessels (G) and cyan fluorescent *claudin 5a* (H). Boxed areas are shown enlarged in I and J, showing high magnification of the network formed by *claudin 5a*-expressing cells forming the mCP. Scale bars: 100 μm in A; 50 μm in B,C; 10 μm in D,E; 50 μm in F-H; 10 μm in I,J.

The CP is a contiguous epithelial sheet with tight junctions (Lun et al., 2015). To further validate our transgenic, and demonstrate its utility in studying the fine structure of the CP, we labelled endogenous protein via immunohistochemistry with a monoclonal antibody to mammalian claudin 5 for comparison. Antibody labelling identified a tight network of epithelial cells with claudin 5 localized to the cell margins in both the mCP and dCP structures (Fig. 6A-E). This correlated with *cldn5a*:EGFP expression in the *TgBAC(cldn5a:EGFP)^vum1^* and light-sheet imaging was able to resolve the same network of cells and cell junctions, even though the subcellular localizations are not exactly the same (Fig. 6F-J). Differences in localisation of EGFP expression were found, due to accumulation of expressed protein in the cytoplasm of cells in the *TgBAC(cldn5a:EGFP)^vum1^* transgene and localisation of antibody labelling at the cell membranes.

### The *cldn5a*:EGFP-expressing sheet contains ciliated ependymal cells that drive cerebral spinal fluid flow

**Fig. 7.**
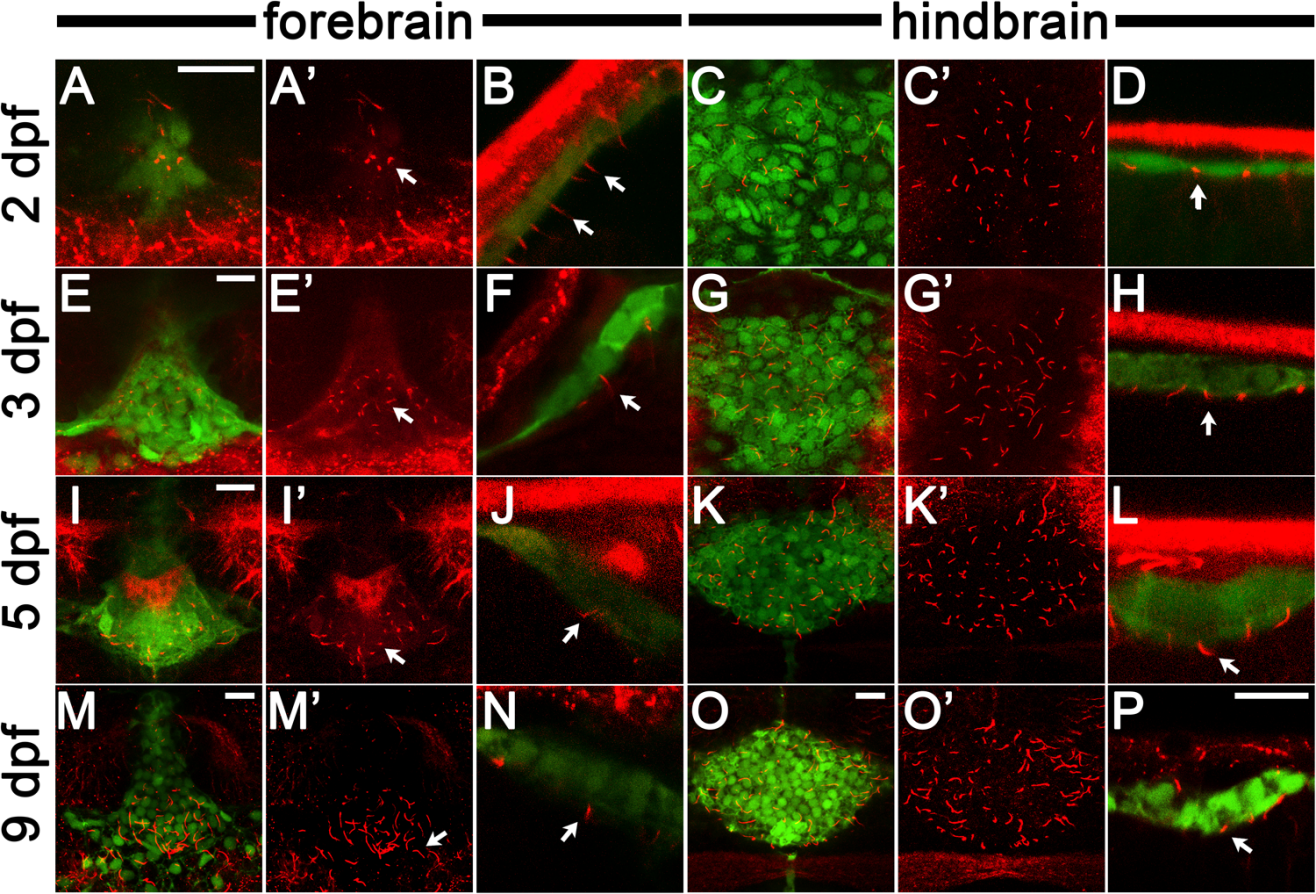
***cldn5a*:EGFP-labelled cells in the** CP **are ciliated throughout development.** Images of cilia visualized by staining with anti-glutamylated tubulin antibody (red) and *cldn5a:EGFP*-positive cells (green). (A,E,I,M) Confocal images of the dorsal view of the dCP in 2, 3, 5, 9 dpf as indicated. (B,F,J,N) Side views of the dCP at the same stages. Cilia project from GFP-positive cells. (C,G,K,O) Dorsal views of the mCP at 2, 3, 5, 9 dpf, respectively. (D,H,L,P) Side views of the mCP. Cilia projected from the GFP-labelled mCP cells. For A,C,E,G,I,K,M,O, the red channel is shown separately to the right. Arrows indicate cilia. In dorsal view panels, anterior is down. In all side view panels, dorsal is up. Scale bars: 15 µm. Scale bar in P applies to all side view images (B,D,F,H,J,L,N,P). Scale bar in O applies to all dorsal views of the mCP (C,G,K,O).

The cells of the CP are a specialized type of ependymal cells, which line the brain ventricles. To confirm the identity of our *cldn5a*:EGFP cells we stained for glutamylated tubulin to label cilia. We could image single cilia from *cldn5a*:EGFP cells in the mCP and dCP as early as 2 dpf (Fig. 7A-D). Only monociliated cells were found at all stages examined in the fore and hind brain (Fig. 7A-P). We could determine the polarity of the *cldn5a*:EGFP cells on the basis of the abundant labelling of glutamylated tubulin in the skin, which revealed that cilia project into the brain ventricles (Fig. 7B,D,F,H,J,L,N,P). CSF is under constant flow that is thought to result from a combination of secretion of CSF from cells of the CP and the beating of the cilia lining the brain ventricles (Kramer-Zucker et al., 2005; Sawamoto et al., 2006). Using injection of fluorescently labelled beads we were able to observe vigorous fluid flow in the CSF in both the fore- and hindbrain ventricles (Movies 5 and 6).

## DISCUSSION

Claudin 5 as a prominent TJ protein is a consistent feature between the BBB and blood-CSF barrier (Bill and Korzh, 2014). Here we have used this feature to create an *in vivo* model for real-time analysis of the development, structure and function of the BBB and CP by generating a transgenic zebrafish line that expresses EGFP under the *claudin 5a* promoter. The high homology and synteny with human, the conservation along the teleost lineage and the previous characterisation of Claudin 5a in zebrafish makes *cldn5a* a logical candidate (Abdelilah-Seyfried, 2010; Xie et al., 2010; Zhang et al., 2012).

We show that developmental expression of *cldn5a*:EGFP is restricted to, and starts in both CPs and the midline at 1 dpf, thereby narrowing down the previously shown whole-mount *in situ* hybridizations (Zhang et al., 2010). The presence of Claudin 5a at the CPs at 1 dpf coincides with the inflation of the ventricles (Zhang et al., 2010, 2012) and corroborates its role in this process. Claudin 5a is crucial for tightening the neuroepithelial paracellular barrier, and probably also important for proper formation of the CP, allowing the production of cerebral spinal fluid (CSF) and inflation of both ventricles. These ventricles are connected and form a system through which continues flow of CSF is ensured (Turner et al., 2012). Expression of *claudin 5a* possibly outlines the entire ventricular system, which can be an explanation of the midline staining we observe. Expression in the brain vasculature is only found at 3 dpf.

Within the functional highly diverse CNS, the microvasculature is expected to consist of a heterogeneous population of brain microvascular endothelial cells (BMECs) (Wilhelm et al., 2016). A considerable majority of CNS microvasculature comprise capillaries, of which the BMECs preferentially express genes related to transport of ions and nutrients (Macdonald et al., 2010). BMECs of venules instead show higher expression of genes involved in inflammatory-related processes and were shown to have a looser organisation of tight junctions as compared to capillaries. This suggests a vessel-specific unique role in physiology and pathophysiology (Macdonald et al., 2010). It is likely that the majority of expression of tight junction-related genes cover all vessel types to sustain the protective function of the BBB. Therefore, the observation made in this study that some blood vessel segments lack *claudin 5a* expression was highly surprising. In mice, similar heterogeneous expression of claudin 5 has been observed in the spinal cord, with highest expression in capillaries and small venules and less expression in larger venules (Paul et al., 2013). Induction of experimental autoimmune encephalitis (EAE) led to loss of claudin 5 expression specifically in venules, suggesting an important vessel specific role for claudin 5 in this condition (Paul et al., 2013). Another plausible explanation for the variation in Claudin 5a presence in our model is the anatomical localisation of the blood vessels in respect to brain tissue. Blood vessel segments lacking *claudin 5a* expression were all located at the borders of the brain and in close proximity to meninges. Therefore, it is likely that these vessels are located outside the parenchyma and do not possess a BBB.

Development of the CNS vascular network involves complex changes in endothelium and surrounding tissue and the timing of BBB formation in this process is difficult to pinpoint (Malinovskaya et al., 2016). Elaborate studies in rodents and zebrafish have shown that CNS vascularisation during development mainly occurs through angiogenesis derived from the perineural vascular plexus driven by VEGF and CNS-specific Wnt/beta-catenin signalling (Blanchette and Daneman, 2015; Hagan and Ben-Zvi, 2015; Obermeier et al., 2013; Umans et al., 2017). Within a few days after initiation of vessel formation, restricted properties have been demonstrated by exclusion of fluorescent dyes from the CNS. Remarkably, this seem to happen before astrocyte generation and ensheathment of vessels occur, while these events have always been considered to be essential for BBB establishment (Blanchette and Daneman, 2015). Recently, within a transgenic zebrafish model for BBB development, it was observed for the first time that barriergenesis occurs simultaneously with CNS angiogenesis (Umans et al., 2017). Moreover, expression of TJ proteins is present at the initiation of angiogenesis in the CNS of mice with subsequent increase of TJ functionality during embryogenesis (Daneman et al., 2009, 2010). In the opossum, it has been demonstrated that newly formed blood vessels possess functional properties from their initiation (Ek et al., 2006). Our study offers a possible mechanism for this, whereby new vessels express *claudin 5a* immediately to form TJs, reflecting developmental steps observed in other models and demonstrating how our transgenic will enable determination of TJ and BBB specification and functionality.

The timespan between initial TJ expression and a functionally intact BBB was for long believed to be the main reason for differences in BBB permeability at different ages. However, considering that these events coincide, an alternative explanation is a prolonged permeability of the barrier between blood and CP (Ek et al., 2006; Saunders et al., 2012). The last decade it has become clear that the blood-CP barrier has more functions than solely CSF production and makes significant contributions to brain homeostasis. Junctional, enzymatic and transporter proteins have been identified and the CP may serve as entry route for immune cells, compounds and even pathogens (Lun et al., 2015). The blood-CP barrier is formed by a monolayer of cuboidal epithelial cells, i.e. ependymal cells, which surround stromal tissue and capillaries, and are joined together by tight junctions (Lun et al., 2015). Studies with enhancer trap lines were the first to describe the two CPs in zebrafish and suggested that at least four different cell lineages develop into stromal, epithelial, endothelial and astroglial components (Bill and Korzh, 2014). The previously described enhancer trap line shows a broad expression profile, of an unidentified gene, in the region of the CP (Bill et al., 2008; García-Lecea et al., 2008). In precision, our transgenic line corroborates and extends those findings and shows cells originating from the roofplate express *claudin 5a* and develop into ciliated ependymal cells.

The treatment of CNS disease is severely impeded by an inability to modulate the entry and exit of therapeutic compounds. Therefore, an improved understanding of the biology of the BBB and blood-CP barrier is of essential importance to improve treatment. Claudin 5 is a tempting target for manipulation, since many hydrophilic drugs prefer to cross the BBB via the paracellular pathway (Greene and Campbell, 2016; Saunders et al., 2013). However, to reduce potential dangerous consequences extensive studies in *in vivo* models are needed before this therapy can be applied in the clinical setting (O'Keeffe and Campbell, 2016). Promising results were achieved in a mice model for cerebral oedema, a major cause for morbidity and mortality in a wide variety of conditions, such as severe traumatic brain injury, neurological cancers and brain infections. Strategies to prevent or treat this condition are limited, but transient modulation of claudin-5 with RNAi led to reduced brain swelling and a better outcome (Campbell et al., 2012). In zebrafish, a mutated fragment of clostridium perfringens enterotoxin has been shown to specifically target and modulate claudin 5, inducing transient paracellular permeability of the BBB (Liao et al., 2016). As zebrafish have also proven to be an outstanding model for new compound screens (North et al., 2007; Robertson et al., 2014), analysis of pharmacodynamics of the compound entry into the CNS is now eminently feasible.

## MATERIALS AND METHODS

### Identification of zebrafish Claudin 5 and BAC recombineering

Human (genome build GRCh38.p7) CLDN5 protein sequence (NP_003268.2) was used in a BLAST search of the zebrafish protein database (genome build GRCz10). Zebrafish Claudin 5a and Claudin 5b proteins were identified and aligned with human claudin 5 using Clustal Omega. Using synteny and expression pattern zebrafish *claudin 5a* was confirmed as the homologue of human *CLDN5*. A search of zebrafish genome BACs identified BAC 187M8 from the CHORI211 library (Robert Geisler and Pieter de Jong, Children's Hospital Oakland Research Institute) as suitable for generation of a fluorescent reporter line due to significant flanking sequence up- and downstream of the *claudin 5a* gene. Primers were designed with a forward primer with 50 bp upstream and including the ATG codon of *claudin 5a* and 24 bp of the targeting vector containing EGFP and a Kanamycin resistance cassette (Dee et al., 2016). The reverse primer contained the reverse complement sequence of the 50 bp downstream of the ATG codon of *claudin 5a* and the reverse complement of the end of the cassette sequence. Forward primer: AACTTCTAAACTCCTTTTAGTACCATCAGGAGTGGGAAAAGAAAGCGATGGTGAGCAAGGGCGAGGAGCTGTTC; reverse primer: GTCCCGCAGACGCACAGGATCAGACCCAGGAGCTCCAAAGCCGCGGAGGCGATATCTGCAGAATTCGCCCTTGA. Tol2 homology arms (Fig. S1) were added as described previously (Gray et al., 2011). Two nanolitres of recombineered BAC DNA at a concentration of 50 ng/µl combined with itol2 mRNA (Abe et al., 2011) at 30 ng/µl. were injected to perform transgenesis.

### Zebrafish

Maintenance of adult zebrafish took place at 26°C in aerated 5 litre tanks, in a 10:14 h light:dark cycle. Eggs were collected within the first hour postfertilization and injected at the 1-4 cellular stage. Injection was performed as described previously (Benard et al., 2012). Initial transgenesis was performed on: (1) WT zebrafish (van der Sar et al., 2004); (2) casper zebrafish, transparent because these zebrafish lack pigment (White et al., 2008); and (3) *Tg(kdrl:mCherry)*, with red fluorescent endothelial cells (Jin et al., 2005). All procedures involving zebrafish embryos and larvae and adult were performed in compliance with local animal welfare laws under Dier Ethische Commissie (DEC) protocol (MMI 12-01).

### Transgenesis

At 4 dpf, larvae injected with the construct were analysed for transgenic expression with a Leica MZ16FA fluorescence microscope. F0-embryos expressing EGFP in the brain region were selected and grown until reproducing age. Subsequent selection took place and F1 larvae with good expression were used for egg production. F2 larvae were used for further analysis and experiments described here. Stable germline transgenics *TgBAC(cldn5a:eGFP)^vum1^* and *Tg(kdrl:mCherry)^is5^;TgBAC(cldn5a:eGFP)^vum2^* were generated and used for the experiments.

### Whole-mount zebrafish larval staining

Visualisation of Claudin 5 expression in the BBB of zebrafish larvae was done by performing whole-mount immunohistochemical staining on fixed larvae. For this, larvae were euthanized at indicated time points with tricaine (E10521, Sigma-Aldrich) and fixed in 4% (V/V) paraformaldehyde/PBS (100122, Electron Microscopy Sciences, Hatfield, USA) at 4°C overnight or at room temperature (RT) for 4 h in microfuge tubes. Fixed larvae were dehydrated and stored in 100% methanol at −20°C until anti-claudin 5 staining was performed. In short, larvae were rehydrated, rinsed with 1% PBTx (PBS+1% Triton X-100), permeated in 0.24% trypsin in PBS and blocked for 3 h in block buffer [10% normal goat serum (NGS) in 1% PBTx (V/V)] Incubation with the primary antibody was performed overnight at RT [mouse anti-Claudin 5 (4C3C2), 187364, Invitrogen; 1:500 dilution] in antibody buffer [PBTx containing 1% (V/V) NGS and 1% (W/V) BSA]. After washing again with PBTx and incubation for 1 h in block buffer, embryos were incubated in the secondary antibody (goat anti-mouse Alexa-647, A21070, Invitrogen; 1:400 dilution), overnight at 4°C. Embryos were then washed with PBTx 5 times, 10 min each.

For staining with anti-glutamylated tubulin, washing with PBST (PBS+0.1% Tween) was applied after fixation. Samples were transferred to 1% Triton X-100 in PBST and incubated at RT for 1-5 days to permeabilize. Larvae were placed in the blocking buffer [0.5% (V/V) Triton, 2% (V/V) normal goat serum in PBST] at room temperature for 2 h. Subsequently, the blocking buffer was removed and replaced with blocking buffer containing the primary antibody [anti-glutamylated tubulin (GT335) mouse IgG, AdipoGen Life Sciences, Liestal, Switzerland; 1:650 dilution]. Specimens were incubated in this solution at 4°C overnight. Larvae were then washed in PBST three times, 30 min each, on a rotator and incubated in secondary antibody (goat anti-mouse Alexa-568, Invitrogen; 1:500 dilution) in the blocking buffer for 4 h at room temperature. Embryos were washed with PBST three times, 10 min each.

### Microscopy

For imaging, embryos were mounted in a drop of 1.5% low melting agarose placed on the surface of 1% agarose gel layer in a 35-mm petri dish [A9414, Sigma-Aldrich (now Merck, Darmstadt, Germany)] or embedded in 1% low melting-point agarose [12841221-01, Boehringer Mannheim (Roche Diagnostics), Basel, Switzerland] dissolved in egg water (60 μg/ml instant ocean see salts) in an eight-well microscopy μ-slide (http://www.ibidi.com). Analysis was performed with a confocal laser scanning microscope (confocal, Leica TCS SP8 X; microscope, Leica DMI 6000). LAS software and ImageJ software were used to generate 3D models, adjust brightness and contrast and create overlays. Adult zebrafish were euthanized with tricaine (E10521, Sigma-Aldrich) and directly embedded in 2% low melting-point agarose (12841221-01, Boehringer Mannheim) dissolved in egg water (60 μg/ml instant ocean see salts) with the dorsal side up. The cranial roof was removed to expose the brain. Analysis was performed with a Leica MZ16FA Fluorescence Stereo Microscope. Brightfield and fluorescence images were generated with a Leica DFC420C camera using LAS software.

### Time-lapse imaging

Time-lapse imaging was performed with light-sheet fluorescence microscopy on a Zeiss Z1 with Z.1 detection optics 20×1.0 NA water immersion objective lens. Zebrafish larvae were mounted in 0.8% low melting point agarose (A9414, Sigma-Aldrich) in E3 containing 0.168 mg/ml tricaine (E10521, Sigma-Aldrich). Z-stacks were captured every 30 min over 12.5 h or 16 h. Maximum intensity projections were generated in Zeiss Zen software. Image processing (cropping, generation of merged images, and linear adjustment of pixel levels) was performed in Fiji ImageJ 2.0.0. Tracking was performed using the Manual Tracking plugin included in Fiji.

### Bead flow assay

Nacre fish were injected with 4×10^3^ 1.75 mm beads (fluoresbrite carboxylate; Polysciences Inc., Warrington, USA) into the hindbrain ventricle 2 dpf. Bead flow was imaged 24 h postinfection in the hind- and forebrain ventricles with a Nikon Ti-E with a CFI Plan Apochromat λ 20×, 0.75 NA objective lens, and using Intensilight fluorescent illumination with ET/sputtered series fluorescent filters (Chroma, Bellow Falls, VT, USA). Images were captured with Neo sCMOS, 2560×2160 Format, 16.6 mm×14.0 mm Sensor Size, 6.5 μm pixel size camera (Andor, Belfast, UK) and NIS-Elements (Nikon, Richmond, UK). Images were processed (cropping, contrast enhancement) using NIS-Elements.

## Supplementary Material

Supplementary information
