# Supplementary material for: A transgenic zebrafish model for the in vivo study of the blood and choroid plexus brain barriers using claudin 5
Source: Biol Open. 2018 Feb 15;7(2):bio030494. doi: 10.1242/bio.030494 (PMC5861362; doi:10.1242/bio.030494)
Supplement: Supplementary information [file biolopen-7-030494-s1.pdf]

Supplemental Material

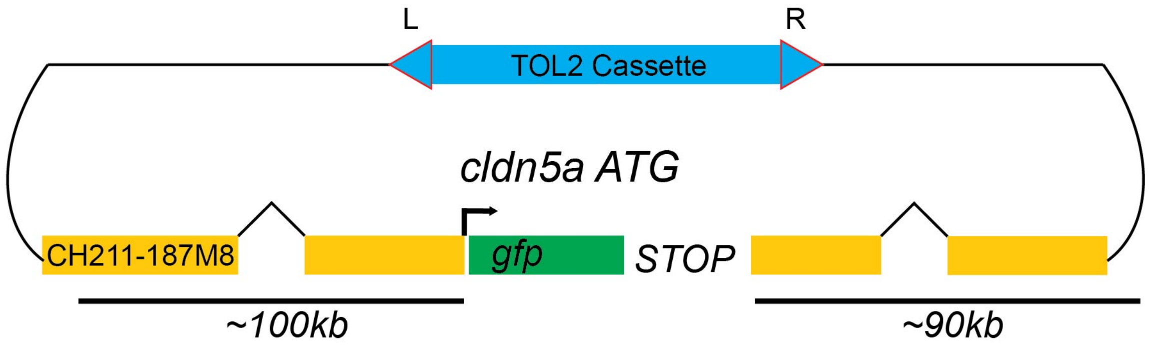

Figure S1: BAC recombineering

Illustration of BAC recombineering strategy. CHORI211 library BAC 187M8 was targeted with the insertion of eGFP following the ATG of the claudin5a gene. Tol2 recombination sites were then added to increase likelihood of stable genome integration.

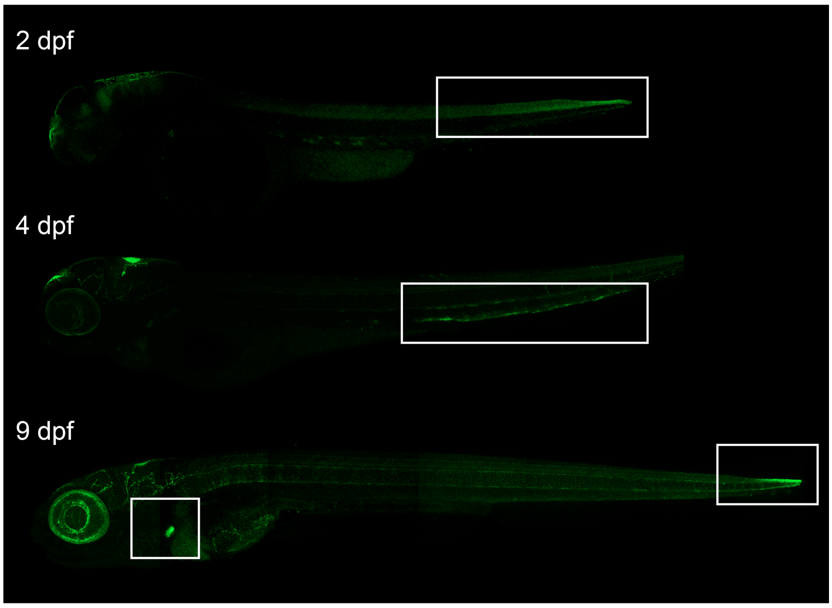

Figure S2: Extra-neuronal regions with transient expression of *Claudin5*

Sagittal images of Tg(cldn5a:GFP) larvae at different time points showing transient expression at other locations than the central nervous system. Expression was observed in the caudal hematopoietic tissue (CHT) between 2-4 dpf, in the tip of the tail and in the heart region at 9 dpf.

Biology Open • Supplementary information

Movies

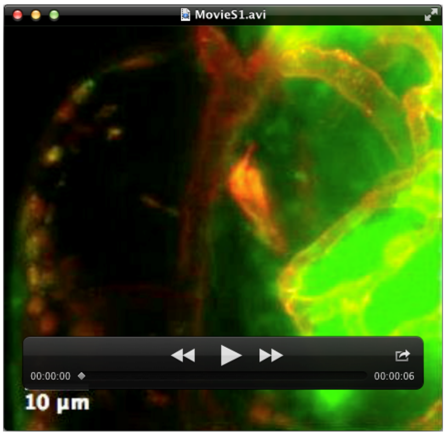

Movie S1 – Angiogenesis 1

Blood vessel development in *Tg(kdrl:mCherry)*^{is5};*TgBAC(cldn5a:EGFP)*^{vum2} larva at 96 hpi. Orientation: dorsal view, blood vessels in the optic tectum of the midbrain. Time lapse made over 12.5 hours. Corresponding still images can be found in Figure 4A.

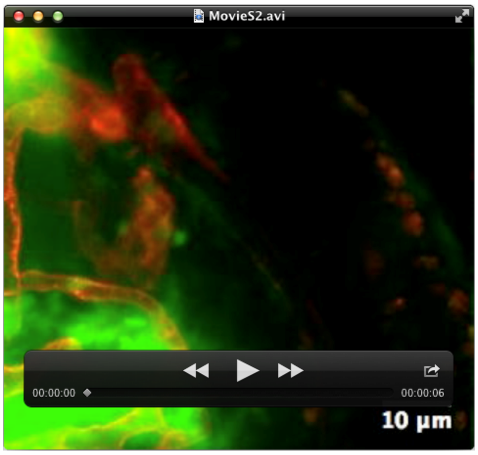

Movie S2 – Angiogenesis 2

Blood vessel development in *Tg(kdrl:mCherry)*^{is5};*TgBAC(cldn5a:EGFP)*^{vum2} larva at 96 hpi. Orientation: dorsal view, blood vessels in the optic tectum of the midbrain. Time lapse made over 12.5 hours. Corresponding still images can be found in Figure 4B.

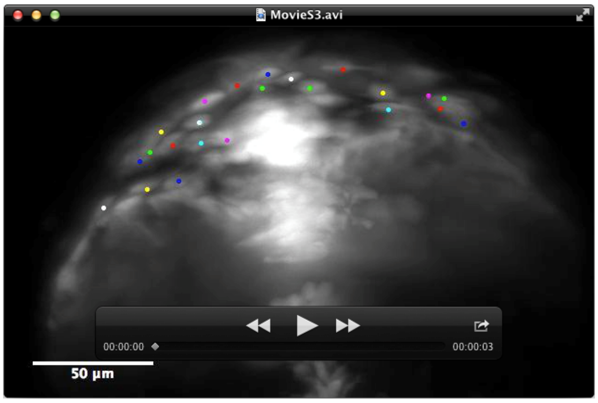

Movie S3 – Cell tracking dCP development

Cell tracking of *Cldn5a:EGFP* positive cells in the forebrain ventricle demonstrating that this structure formed via collective cell migration. Z-stacks were captured every 30 minutes over 16 hours between 2 and 3 dpf. Corresponding still images are presented in Figure 5E.

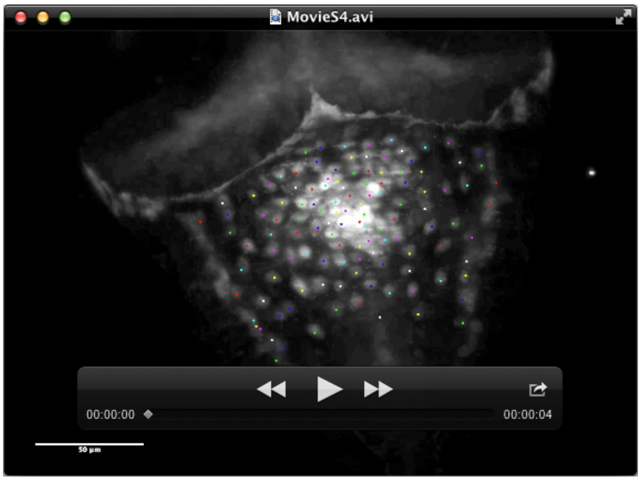

Movie S4 – Cell tracking mCP development

Cell tracking of *Cldn5a:EGFP* positive cells in the forebrain ventricle demonstrating that this structure formed via collective cell migration. Z-stacks were captured every 30 minutes over 16 hours between 2 and 3 dpf. Corresponding still images are presented in Figure 5F.

Biology Open • Supplementary information

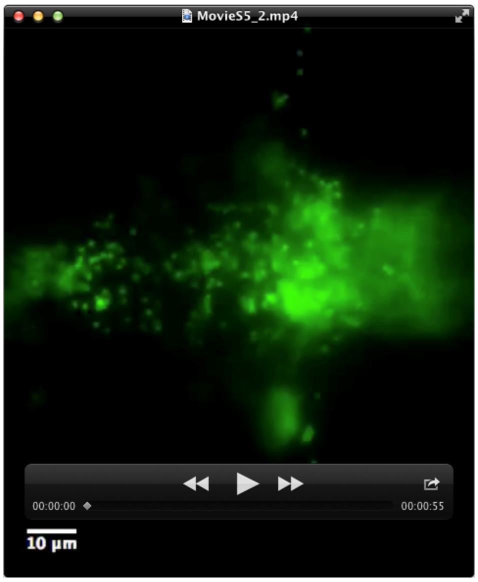

Movie S5 – ventricular circulation, beads, dCP

CSF flow in the forebrain ventricle visualized by time lapse imaging of fluorescent beads. Dorsal view with the anterior of the larvae to the left, posterior to the right. Images were captured every 200 ms and presented at 50 frames per second.

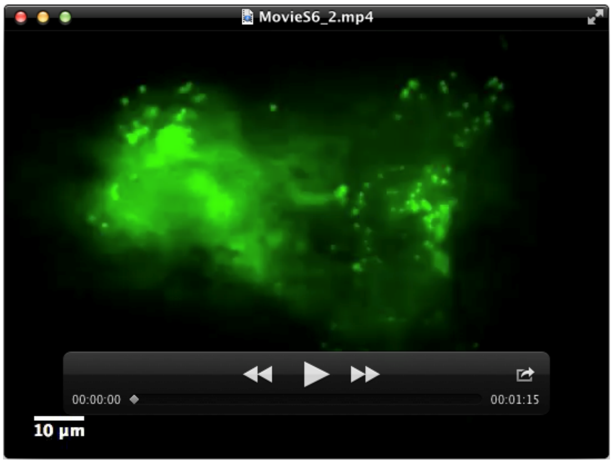

Movie S6 – ventricular circulation, beads, mCP

CSF flow in the hindbrain ventricle visualized by time lapse imaging of fluorescent beads. Dorsal view with the anterior of the larvae to the left, posterior to the right. Images were captured every 200 ms and presented at 50 frames per second.

Biology Open • Supplementary information
